# Uptake of PMTCT sites for increasing accessibility of services in prevention of mother to child HIV transmission program in Rwanda, January 2005 June 2010

**DOI:** 10.1186/1742-4690-9-S1-P100

**Published:** 2012-05-25

**Authors:** Ange Anitha Irakoze, Placidie Mugwaneza, Sabin Nsanzimana, Jennifer Mbabzi, Jean Pierre Nyemazi, Eric Remera

**Affiliations:** 1 Hiv Division, Former Trac Plus, Kigali, Rwanda

## Background

In Rwanda, the Prevention of Mother to Child Transmission of HIV program (PMTCT) started in 1999 as a pilot project; a positive evaluation conducted 1 year later lead to national scale-up. The integration of the PMTCT program into existing antenatal care (ANC) services was done gradually. Nationally, HIV testing is routinely offered to all pregnant women at the time of enrollment in ANC. Political commitment and involvement of key stakeholders in program implementation; public awareness campaigns, especially for pregnant women; and the involvement of local authorities, care providers, and community health workers have greatly contributed to the scale-up of the PMTCT program in Rwanda.

## Methods

We analyzed routinely reported facility-level data on HIV testing among pregnant women in the national PMTCT program from January 2005 to June 2010.

## Results

The number of PMTCT sites increased from 209 (46%) at the end of 2005 to 382 (84%) sites in June 2010 (209 in 2005, 234 in 2006, 285 in 2007, 342 in 2008, 377 in 2009 and 382 June 2010) out of 452 health facilities providing ANC services. Overall, 1,623,079 women received ANC services; of these, 1,554,387 (95.76%) accepted HIV testing. The acceptance rate of HIV testing among pregnant women in ANC increased from 89% in 2005 to 98,3% in June 2010. Of women tested, 99,4 % received their HIV test result. HIV prevalence among pregnant women tested decreased from 4.8% in 2005; to 4.4% in 2006; to 3.8% in 2007; to 2.9% in 2008 and 2.6% in 2009 and 2010.

## Conclusions and recommendations

These results demonstrate that many efforts were done regarding the increasing of PMTCT sites in the line of scale-up of the national PMTCT program in Rwanda, as indicated by increases in number of sites offering PMTCT services, in uptake of HIV testing for pregnant women. Despite the high uptake of PMTCT sites, it is still below the national target of 100% of coverage of PMTCT sites, thus enhanced efforts to increase availability of PMTCT services are warranted.

